# The Cumulative Incidence of Post-Traumatic Epilepsy After Mild Traumatic Brain Injury: A Systematic Review and Individual Participant Data Meta-Analysis Protocol

**DOI:** 10.1089/neur.2023.0117

**Published:** 2024-05-31

**Authors:** Joshua Z. Goldenberg, Richard Davis Batson, Mary Jo Pugh, Heather Zwickey, Jennifer Beardsley, Maurice P. Zeegers, Michael Freeman

**Affiliations:** ^1^ Helfgott Research Institute, National University of Natural Medicine, Portland, Oregon, USA.; ^2^ Department of Epidemiology, Care and Public Health Research Institute, Maastricht University, Maastricht, the Netherlands.; ^3^ VA Salt Lake City Health Care System, Salt Lake City, Utah, USA.; ^4^ Independent Research Librarian, Seattle, Washington, USA.; ^5^ Forensic Research + Analysis, Portland, Oregon, USA.

**Keywords:** adult brain injury, epilepsy, head trauma, post-traumatic seizure, traumatic brain injury

## Abstract

A precise understanding of the latency to post-traumatic epilepsy (PTE) following a traumatic brain injury (TBI) is necessary for optimal patient care. This precision is currently lacking despite a surprising number of available data sources that could address this pressing need. Following guidance from the Cochrane Collaboration and Joanna Briggs Institute, we conduct a systematic review to address the research questions: What is the cumulative incidence of PTE following mild TBI (mTBI; concussion), and what is the distribution of the latency to onset? We designed a comprehensive search of medical databases and gray literature sources. Citations will be screened on both abstract and full-text levels, independently and in duplicate. Studies will be evaluated for risk of bias independently and in duplicate using published instruments specific to incidence/prevalence studies. Data will be abstracted independently and in duplicate using piloted extraction forms. Disagreements will be resolved by consensus or third-party adjudication. Evidence synthesis will involve pairwise and individual participant data meta-analysis with heterogeneity explored via a set of predetermined subgroups. The robustness of the findings will be subjected to sensitivity analyses based on the risk of bias, outlier studies, and mTBI definitional criteria. The overall certainty in the estimates will be reported using GRADE (Grading of Recommendations, Assessment, Development, and Evaluations). This protocol presents an innovative and impactful approach to build on the growing body of knowledge surrounding post-mTBI PTE. Through a precise understanding of the latency period, this study can contribute to early detection, tailored interventions, and improved outcomes, leading to a substantial impact on patient care and quality of life.

## Background and Rationale

Traumatic brain injury (TBI) is relatively common leading to dramatic disability and socioeconomic burden worldwide. Global estimates suggest that there are 69 million TBIs per year, with an annual U.S. incidence of 1,300 per 100,000 individuals.^1^ The severity of TBI is classified as mild, moderate, or severe. Mild TBIs (mTBI), also known as concussions, make up 80% of all TBIs.^2^ mTBI is defined by the American Congress of Rehabilitation Medicine^3^ as follows:

*A traumatically induced physiological disruption of brain function as manifested by at least one of the following: 1. any period of loss of consciousness; 2. any loss of memory for events immediately before or after the accident; 3. any alteration in mental state at the time of the accident (e.g., feeling dazed, disoriented, or confused); and 4. focal neurological deficit(s) that may or may not be transient; but where the severity of the injury does not exceed the following: loss of consciousness of approximately 30 minutes or less; after 30 minutes, an initial Glasgow Coma Scale of 13–15; and post-traumatic amnesia not greater than 24 hours.*

Seizures following a TBI are categorized as either early (within seven days of injury) or late (more than seven days). Those only occurring early are generally not considered epilepsy. Post-traumatic epilepsy (PTE), in contrast, is a seizure disorder defined as unprovoked seizures >7 days after a TBI.^4^ It is a well-documented TBI complication with a reported prevalence of 1% to 53%, depending on the severity of the initial brain injury^5,6^ and the follow-up time of the study. PTE can have dramatic impacts on one’s quality of life, and carries significant financial burdens, with annual direct cost estimates in the United States as high as $20,000.^7^

In the preliminary work for this review, we identified 14 PTE incidence/prevalence studies reporting on mTBI specifically. The seminal study^5^ on PTE in an mTBI population used data from the prospective Rochester Epidemiology Project, a 50-year cohort study in a defined population in Olmsted County Minnesota. In that study, Annegers and colleagues reported an incidence rate ratio of 17.0 for severe TBI, 2.9 for moderate TBI, and 1.5 for mTBI. While their report suggests a prevalence of 1% for mTBI PTE, other studies have reported estimates almost four times higher.^8^ The varied prevalence estimates in mTBI may relate to differences in how PTE is diagnosed, the baseline population, length of follow-up, and the inclusion of complicated versus uncomplicated mTBI. A systematic review could be inclusive of varied study environments and clinical scenarios, yet allow for rigorous heterogeneity exploration (i.e., subgroup effect testing).^9^ However, we are aware of no systematic review on the prevalence of PTE post-mTBI.

Most PTE occurs after a latency period, during which a process of epileptogenesis takes place that involves structural, molecular, and functional changes in the brain.^10^ While current research describes the latency periods as variable and suggests that 40% of PTE will occur within six months,^11^ a more precise incidence distribution is lacking in mTBI specifically, despite a surprising number of available data sources that could address this evidence gap.

While injury-to-seizure latency information was presumably recorded in the 14 identified studies mentioned above, it was not reported, leaving these essential data inaccessible to consumers of the literature. Another source of data that is currently inaccessible is the mTBI-specific data from studies that included TBI survivors across all severity levels. Individual participant data meta-analysis (IPDMA) could leverage available but currently inaccessible data to elucidate the latency distribution of PTE in mTBI, and maximize the precision of prevalence estimates. We are aware of no such analysis published to date.

Patients, clinicians, and scientists will be better served by a systematic review of all available prevalence estimates of PTE after mTBI, providing insight into the latency distribution of PTE in this population and exploring relevant heterogeneity. Importantly, this may be achievable with obtainable data sources.

The specific questions this systematic review addresses are as follows: (1) what is the cumulative incidence of PTE after an mTBI and (2) how is this cumulative incidence distributed over time (i.e., what is the latency distribution)?

### Eligibility criteria

- Population: Adult humans who have experienced an mTBI at any point in their history
  ∘ “Adult” will be as defined by the primary study authors but if <80% of the participants are 18 years of age or older, this will be noted.
  ∘ mTBI will be defined by study authors but differences in definition will be noted. mTBI with or without loss of consciousness will be included.
  ∘ Studies where mTBI data are reported among other TBI severities will be included if the mTBI-specific data are obtainable.
  ∘ Exclusion: Animal studies, pediatric populations, TBI of severity levels moderate or severe, TBI where severity levels are not defined, and mTBI-specific data are not obtainable.

- Condition: PTE
  ∘ As defined by study authors (e.g., using ICD codes, chart review).
- Exclusion: Studies that include seizures occurring exclusively within 7 days of the mTBI (early seizures) will be excluded unless data on PTE prevalence exclusively defined as seizures occurring >7 days after the mTBI are obtainable (e.g., reported subgroup analysis, unpublished data from contact with authors).

- Context: Studies from all nations, of all ethnicities, and both civilian and military populations will be included. TBIs from all causes (e.g., motor vehicle accidents, sports injuries, blast injuries, falls) will be included.
- Types of studies: observational studies (e.g., cross-sectional surveys, cohorts) and the control arms of randomized controlled trials
  ∘ The control arm of randomized trials of mTBI patients will be used for PTE frequency data (if reported) if the control arm is usual care, waitlist, or placebo/sham.
- Excluded: randomized controlled trials with only active controls.

## Information Sources/Search Strategy

A search of medical databases and gray literature sources, including administrative and vital statistics sources, will be conducted. Medical databases will include MEDLINE (PubMed), Embase (embase.com), CINAHL Complete (EBSCO), and the Cochrane Library (CENTRAL). The database search will be augmented by a web-crawler search (Google Scholar), registry searches (Clinicaltrials.gov, etc.), content expert discussion, and citation tracking. Included studies and related reviews will be subject to forward and backward citation tracking. Citation tracking will be conducted by manually reviewing the references of included studies and by using vector score search (PubMed’s Similar Articles Tool) and cocitation and bibliographic coupling (connected papers.com) of the identified “seed” articles. Citation tracking will be iteratively conducted until no new relevant articles are identified.

Administrative and vital statistics data sources will include the National Center for Health Statistics, the Medical Expenditure Panel Survey, the National Health Interview Survey, and the National Health and Nutrition Examination Survey. In addition, the research teams of large TBI studies (TRACK TBI, CENTER TBI, LIMBIC/CENC) will be contacted and queried about relevant published and unpublished data.

Search strings were built upon text words and, where relevant, subject heading terms (e.g., Medical Subject Heading-MeSH, Emtree terms), based on the core search terms of “concussion,” “traumatic brain injury,” “epilepsy,” “seizures,” and “post-traumatic seizures.” The search string was initially built for PubMed (Table 1) but then translated using the Polyglot Search Translator.^12^ The translations and the entire search strategy were designed by a medical librarian trained and experienced in the conduct of systematic review searches. The search strategy was subjected to further independent review following the guidance of electronic search PRESS peer review.^13^ No language or date restrictions were used. No restrictions on peer review or publication status will be placed (e.g., posters, abstracts, dissertations), although these evidence sources will be differentiated in the evidence summary. An animal study exclusion filter was used.

**Table 1.** PubMed Line-by-Line Search Strategy

#1	Epilepsy, Post-Traumatic[mh] 1,306
#2	epilep*[tiab] 166,110
#3	seizures[mh] 73,949
#4	seizure*[tiab] 144,715
#5	convuls*[tiab] 32,016
#6	#1 OR #2 OR #3 OR #4 OR #5 260,971
#7	Brain Injuries, Traumatic[mh] 24,510
#8	“Brain injury”[tiab:∼3] 88,265
#9	“Brain injuries”[tiab:∼3] 12,343
#10	TBI[tiab] 31,955
#11	mTBI[tiab] 4,097
#12	Head Injuries, Closed[mh] 15,368
#13	concuss*[tiab] 13,124
#14	“Brain trauma”[tiab:∼3] 5,169
#15	“Brain traumas”[tiab:∼3] 121
#16	“Brain traumatic”[tiab:∼3] 51,026
#17	“Head injury”[tiab:∼3] 22,906
#18	“Head injuries”[tiab:∼3] 12,360
#19	“Head trauma”[tiab:∼3] 12,711
#20	“Head traumas”[tiab:∼3] 274
#21	“Head traumatic”[tiab:∼3] 1,735
#22	#7 OR #8 OR #9 OR #10 OR #11 OR #12 OR #13 OR #14 OR #15 OR #16 OR #17 OR #18 OR #19 OR #20 OR #21 144,176
#23	#6 AND #22 7,496
#24	#23 NOT (animals [mh] NOT humans [mh]) 6,317

## Screening

Citations in a format that can be uploaded to a citation manager (e.g., .ris, .nbib) will be screened using Eppi-Reviewer. Studies of interest in languages other than English will be initially translated using Google Translate, and essential key details confirmed, when possible, with fluent speakers of that language. All citations from all databases and each vector score search and bibliography coupling network will be screened, but only up to 200 citations from web crawler searching will be screened as per published guidance.^14^ Screening will first be conducted on a title/abstract level and subsequently reviewed on a full-text level with reasons for exclusion noted. Screening will be done independently and in duplicate. In case of disagreement in which consensus cannot be reached, a senior researcher will serve as an adjudicator. Study flow will be tracked and reported in a PRISMA study flow diagram.^15^

## Data Extraction

Data extraction will be conducted independently and in duplicate with disagreements resolved by consensus or by an adjudicator if needed. A piloted and standardized extraction table using Google Sheets will be used. The extracted information will include the following:

- Size of the total cohort
- Number of those within the total cohort who had an mTBI
- Length of latency (i.e., length of time from mTBI until PTE)
- How the mTBI was defined
- Percentage of those with complicated versus uncomplicated mTBI
- Number of those with mTBI who developed seizure/epilepsy
- How the seizures/PTE were defined
- Civilian versus military population
- Age of the cohort (mean, median, range)
- Length of follow-up (i.e., the observation period post-mTBI where PTE was recorded) Cohort source (national, hospital)

Additionally, the information needed for risk of bias assessment will be extracted. Our primary outcome of interest is the cumulative incidence of PTE.

## Quality Assessment

All studies will be reviewed for risk of bias across 10 domains, covering issues of internal and external validity with each study given a summary score (high/low risk of bias). A peer-reviewed instrument developed by Hoy et al., which is based on the Cochrane Risk of Bias tool, but modified to be directly applicable to incidence/prevalence studies, will be used.^16^

The diagnosis of both PTE and mTBI and can be challenging. For example, functional (nonepileptic) attacks after mTBI may be misdiagnosed as epilepsy, and mTBI may be inappropriately diagnosed based on reports of amnesia, which may be secondary to causes external to the injury in question. Concerns with appropriate diagnosis will be considered in our assessment of domain 6 in the Hoy instrument, which deals with “acceptable case definitions.”

## Evidence Synthesis

Where the identified evidence is sufficiently homogeneous, it will be combined in a quantitative evidence synthesis (meta-analysis). To avoid selection bias and to best explore heterogeneity, the primary analysis will be more inclusive of clinical and methodologic heterogeneity, but heterogeneity will be explored rigorously as described below.

Two evidence synthesis approaches will be used: a pairwise meta-analysis and an IPDMA. A pairwise meta-analysis combines study-level data (i.e., study-level prevalence estimates and their variance), whereas an IPDMA combines participant-level data, while still respecting the integrity of the study-level randomization. Each approach has advantages and limitations and both are needed to best address our research questions. Specifically, while IPDMA improves power, especially in terms of participant-level variables (e.g., latency, age, sex), obtaining participant-level data from researchers is challenging and realistically will limit the includable studies, censoring the available data for analysis. A pairwise meta-analysis therefore may provide the broadest collection of data for study-level variables. However, IPDMA is needed to address our latency question (participant-level data) as well as to allow the inclusion of mTBI data from studies of mixed TBI populations (mTBI, moderate TBI, and severe TBI).

For the pairwise meta-analysis, prevalence estimates will be pooled using a random-effects inverse variance model^17^ applying the metaprop function from the “meta” package^18^ within the coding environment R.^19^ A pooled prevalence with a 95% confidence interval will be generated. Heterogeneity will be measured using the *I*^2^ statistic. Results will be depicted visually in a forest plot.

For the IPDMA, individual participant data from multiple studies will be aggregated utilizing a generalized linear model implemented with the lme4 package within the statistical environment of R.^19,20^ Published guidance on the conduct of IPDMAs will be followed,^21^ and cumulative incidence will be reported over time as a continuous curve as well as pooled 3-month intervals.

## Sensitivity Analysis

Sensitivity analyses will be conducted to explore the potential effects of (1) risk of bias, (2) outlier studies, and (3) mTBI definition. Specifically, we will remove all high-risk of bias studies and observe the effect on the pooled estimate; we will remove single studies iteratively and observe the effect on the pooled estimate; and we will limit analysis to those studies using the American Congress of Rehabilitation Medicine mTBI definition and observe the effect on the pooled estimate. We will conduct tests of interaction on our sensitivity analyses, but our reporting will not be limited to those where the interaction is statistically significant, as these tests are often underpowered. The result of our sensitivity analyses will inform the certainty of evidence assessment (i.e., GRADE), where we may rank down for risk of bias.

## Heterogeneity Exploration

Published guidance from the Cochrane Collaboration will be followed with heterogeneity explored by a limited set of *a priori* subgroups, and when relevant, these will be modified for IPDMA methods (e.g., use of IPDMA-specific quality guidance).^22,23^ In the preliminary work for this project, potential subgroup effects were discussed with the team’s content experts who determined the following *a priori* subgroup analyses as shown in Table 2: age, interval length post-TBI, complicated versus uncomplicated mTBI, population source, military versus civilian, and country income level.

**Table 2.** Heterogeneity Exploration Variables

Variable	Measurement	Hypothesized direction
Age	Mean age; continuous variable	Larger point estimates will be seen in older cohorts
Interval length post-TBI	Mean follow-up period; continuous variable	Larger point estimates will be seen with longer follow-up periods
Complicated vs. uncomplicated mTBI	Dichotomous variable	Larger point estimates will be seen with complicated mTBI
Source population	National registry vs. hospital/clinic cohorts; dichotomous variable	Hospital/clinic cohorts will have higher prevalence estimates
Military vs. civilian population	Dichotomous variable	Military cohorts will have higher prevalence estimates
Country income level	High/low; dichotomous variable	Higher income countries will report a lower prevalence than those in lower income countries

Where primary studies adjust for potential confounders, we will perform a statistical analysis (subgroup test of interaction/meta-regression) in which we will compare adjusted versus not adjusted studies. If this presents evidence of a statistically significant source of heterogeneity, we will present the results in the respective subgroups.

Furthermore, we are planning a concurrent phenomenologically based qualitative study guided by lived experience consultants using semistructured interviews to gain insight into the experiences of individuals with PTE during the latency period. While the primary purpose of this study is to describe the lived experience of these individuals, we will explore the interviews for potential effect modifiers, which may have been recorded, but not reported, in the primary research studies, and use these to inform further *post hoc* subgroup exploration in our IPDMA.

## Quality of the Evidence

The overall certainty (quality) of evidence will be rated using the GRADE (Grading of Recommendations Assessment, Development and Evaluation) approach. GRADE assessments will start with a high level of evidence but will be rated down by one or more levels on the basis of five categories of limitations as follows: risk of bias, inconsistency, indirectness, imprecision, and publication bias.^22^ GRADE will be modified for IPDMA as per standard guidance.^23^ A funnel plot for visual inspection of publication bias will be constructed, and Peter’s statistical test of funnel plot asymmetry (publication bias) will be conducted.^24^

## Potential Impact

### Impact on PTE research

This study provides valuable insights into the incidence and timing of PTE after an mTBI. This understanding will inform future research endeavors, enabling researchers to focus on targeted seizure prophylaxis during the critical latency window between concussion exposure and seizure onset. Moreover, this study contributes to the growing body of knowledge surrounding concussion-related PTE, leading to a more comprehensive understanding of its risk factors, prognostic indicators, and effective management strategies.

### Impact on patient care

This study facilitates the development of evidence-based protocols for clinical follow-up after mTBI. By identifying the latency period during which PTE is most likely to manifest, health care providers can design tailored monitoring plans, conduct appropriate diagnostic tests, and initiate interventions at the right time. This proactive approach may significantly improve patient outcomes, ensuring timely detection and early intervention for PTE. This personalized approach may reduce the burden of PTE, minimize seizure occurrence, and enhance the quality of life for individuals living with PTE.

### Enhancing quality of life

By providing insights into the latency period, this study can contribute to surveillance, early detection, and intervention, allowing individuals to access appropriate health care services promptly. In addition, understanding the latency distribution in PTE following mTBI is vital for planning comprehensive support systems and rehabilitation programs. By tailoring interventions and support services based on accurate data on latency, this study may positively impact the quality of life of these patients.

## Innovation

This research study will be the first comprehensive pairwise meta-analysis as well as the first IPDMA of the cumulative incidence of PTE following an mTBI. While individual primary studies have provided valuable insights, the proposed evidence syntheses will consolidate and analyze existing data in a systematic manner, providing a more robust understanding of the relationship between mTBI and PTE with a particular focus on the latency period. By incorporating individual participant data, we can achieve greater precision and examine potential risk factors, subgroups, and variations in PTE prevalence that may have been overlooked in previous studies.

Another innovative aspect of this study is the utilization of previously unreported mTBI-specific PTE latency data. By collecting and analyzing these data, we can uncover new insights into the time frames in which PTE typically manifests following an mTBI. This information is crucial for surveillance, early detection, intervention, and treatment strategies, ultimately improving patient outcomes and quality of life.

## Conclusion

In conclusion, this protocol presents an innovative and impactful approach to contribute to the body of knowledge surrounding post-mTBI PTE. Through a precise understanding of the latency period, this study can contribute to early detection, tailored interventions, and improved outcomes, leading to a substantial impact on patient care and quality of life.

## Supplementary Material

Supplementary Data S1

## Future Amendments

If future amendments to the protocol are needed, they will be noted in PROSPERO and in the final article.

## Abbreviations Used

GRADE: Grading of Recommendations, Assessment, Development, and Evaluations
IPDMA: individual participant data meta-analysis
mTBI: mild TBI
PTE: post-traumatic epilepsy
TBI: traumatic brain injury
